# Cooking-class style fermentation as a context for co-created science and engagement

**DOI:** 10.1128/spectrum.02660-24

**Published:** 2025-08-15

**Authors:** Hanna L. Berman, Erin A. McKenney, Christina E. Roche, Sarah Michalski, Soo Hee Kwon, Elizabeth Weichel, Amanda Matson, Lauren M. Nichols, Samuel Alvarado, Julie E. Horvath, Robert R. Dunn

**Affiliations:** 1 Department of Applied Ecology, North Carolina State University 733405, Raleigh, North Carolina, USA; 2 Department of Marine, Earth and Atmospheric Sciences, North Carolina State University 6798 , Raleigh, North Carolina, USA; 3 Research and Collections Section, North Carolina Museum of Natural Sciences 41561, Raleigh, North Carolina, USA; 4 Southern Peak Brewery, Apex, North Carolina, USA; 5 Independent Researcher 519780 , Raleigh, North Carolina, USA; 6 Piedmont Picnic Project, Raleigh, North Carolina, USA; 7 Center for Data and Visualization Sciences, Duke University Libraries, Duke University 500033, Durham, North Carolina, USA; 8 Department of Biology, University of West Florida 622575, Pensacola, Florida, USA; 9 Department of Biological Sciences, North Carolina State University 6798 , Raleigh, North Carolina, USA; 10 Renaissance Computing Institute, University of North Carolina at Chapel Hill 427389, Chapel Hill, North Carolina, USA; 11 Office of University Interdisciplinary Programs, North Carolina State University 6798 , Raleigh, North Carolina, USA; University of Mississippi, University, Mississippi, USA

**Keywords:** fermented food, microbiome, participatory science

## Abstract

Fermented foods have been consumed for thousands of years and have been used as a model system to study community succession and other ecological questions. Additionally, cooking classes offer opportunities to learn about food preparation and history. In the present study, scientists and chefs delivered cooking-class style workshops in which participants learned the recipes of one of three fermented foods and the microbial ecology within these foods. Participants prepared jars of chow chow, kimchi, or kombucha to set up experiments to study microbial community succession and pH changes. The fermented foods were also used to test the following hypotheses: that increasing the number of substrates results in increased alpha diversity, and that phylogenetically diverse substrates will lead to greater beta diversity among microbial communities. Microbial communities contained lactic and acetic acid bacteria described previously in fermented foods, and indicator species were identified for cabbage and radish substrates in kimchi. Finally, we qualitatively comment on the experience of developing workshops with chefs and the use of participatory science in these experiments.

**IMPORTANCE:**

The present study demonstrates the utility of using fermented foods as an inexpensive and effective tool to investigate ecological phenomena and engage the public in microbiology and ecology through cooking-class style workshops. We also model a creative, interdisciplinary collaboration between scientists and chefs.

## INTRODUCTION

By now, hundreds of papers have called for more and better engagement among the public, science, and scientists. Often, the response to these calls is to try to find ways for the public to “meet scientists,” where they are. Our experience over the last decade in studying the ecology and evolutionary biology of food, however, suggests an alternative possibility. What if we were to more actively bring science to the public in the context of public interests, questions, and enthusiasm? What would it look like to tailor science itself to the interests of the public? Ultimately, the questions that scientists choose to study are, if not fully arbitrary, heavily influenced by the interests of scientists, human or environmental health, and the cloistered scientific cultures in which they are embedded. As a result, providing the public with more agency in driving the direction of science is, perhaps, a useful complement to existing frameworks for prioritization. Historically, extension systems at land-grant universities have offered a context for the public to guide science. Through these programs, colleges and universities have prioritized extension, or public engagement and outreach, along with teaching and research (1). Faculty at these institutions engaged interested agricultural parties to understand their systems and obstacles and then to perform studies to provide guidance in light of those obstacles. However, this system was imagined in the 1860s, a time when most Americans were farmers, and many of the outreach programs in extension systems have traditionally centered on agricultural themes or practices. Today, fewer than 2% of Americans work on farms (2). Therefore, we sought ways to shift our focus to the urban and suburban areas where the average stakeholder lives and create engaging opportunities that would reflect the populace. In this context, what would it look like to adhere to the mission of extension programs, while also recognizing shifting demographics? We propose that educational outreach and communication centered on food and the science of food offers an opportunity for more and better engagement among the public, science, and scientists. Previously, we have engaged local and global populations through distributed experiments on sourdough bread (3), experiments in person with chefs (4), studying locally milled flour (5), and describing the language and knowledge around sourdough starters in artisan baking culture, home bakers, and food scientists (6). Through these efforts, chefs began to suggest to us another possibility. They noted that members of the public often already gather to learn about food in the context of cooking classes and suggested the possibility of combining cooking classes with hands-on science. Here, we report on such an effort.

Our approach was to structure a series of three cooking classes, focused on fermentation, in which we addressed ecological questions using fermentation. Our aim was to do so in such a way that in making the ferments, participants would also be carrying out replicates of an experiment. Thus, we could simultaneously (i) describe the experimental method, (ii) teach participants how to ferment a particular food, (iii) describe hypotheses about what might be occurring in the experiment, and then (iv) follow up on the experiment to reveal the results. Although our previous fermentation studies involving engagement have focused on sourdough, here, we chose foods that do not require regular feeding and care after the initial recipe. Before going further, we will share two results. First, this was a successful venture with the community. Second, humbly, we note that while the cooking classes took several weeks, the full process of finishing the scientific studies has now taken nearly 7 years. We discuss this temporal mismatch and its challenges in the discussion.

Experimentally, we focused on a series of hypotheses that have been elaborated in experimental outdoor settings, but that have seldom been practically implemented or tested in microbial systems. In other words, we explored the potential of obscure theory to inform our understanding of everyday life. First, we explored the hypothesis that the diversity of resources favors the diversity of consumers. This theory has been well-developed with mathematical models (7, 8). It has also been empirically demonstrated in laboratory contexts (9) and in old fields (10–12). In laboratory experiments of controlled marine microbial environments, increasing the richness of algae species increased the richness of ciliates that consume the algae (9). In field experiments, plots that were experimentally seeded with a higher diversity of plants supported a higher diversity of insect consumers (10). Diversity begets diversity. We tested this hypothesis in a far more practically relevant context, during the production of the fermented relish, chow chow. Working with participants and chefs, we created chow chows that differed in the number of vegetables used in their preparation. Second, we tested a sub-hypothesis of this prediction, namely that closely related plant species should have a weaker effect on microbial diversity than more distantly related plants. We did this by comparing microbial communities of kimchis prepared with one of two different vegetables (napa cabbage, *Brassica rapa* subs. *pekinensis,* and daikon radish, *Raphanus sativus* var. longipinnatus, which diverged ~8 mya [13]) to microbial communities associated with the fermentation of green and/or black tea (different preparations of *Camellia sinensis*). Finally, we hypothesized that pH decreases as richness and diversity increase during succession as ferments mature over time. We have previously demonstrated these phenomena in sourdough (5).

Again, these phenomena have been well-documented in abstract experiments, but less so in useful contexts. In describing our experiments, we include full recipes in the supplement, so that others can repeat these experiments and also make these ferments at home. We also discuss the experience of chefs and scientists collaborating on these workshops and experiments and provide brief qualitative comments on our experience with engaging the public in these workshops.

## MATERIALS AND METHODS

**Fig 1:**
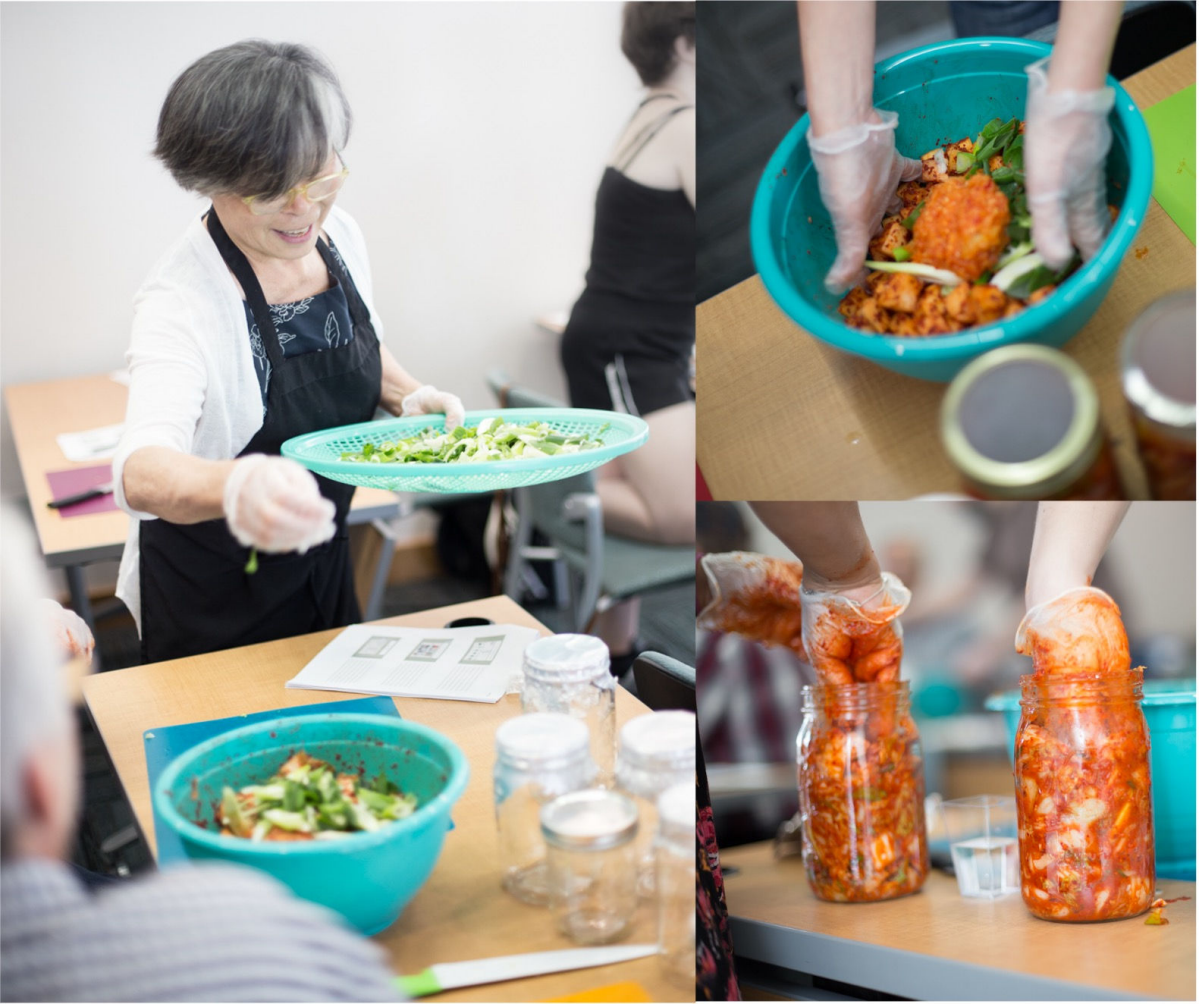
Cooking-class style workshops for experimental setup. Fermented food model ecosystems were set up in workshops where participant scientists prepared fermented foods and learned about the ecology of microbiomes. Here, chef Soo Hee “Mama” Kwon demonstrates kimchi making for participants.

**Fig 2:**
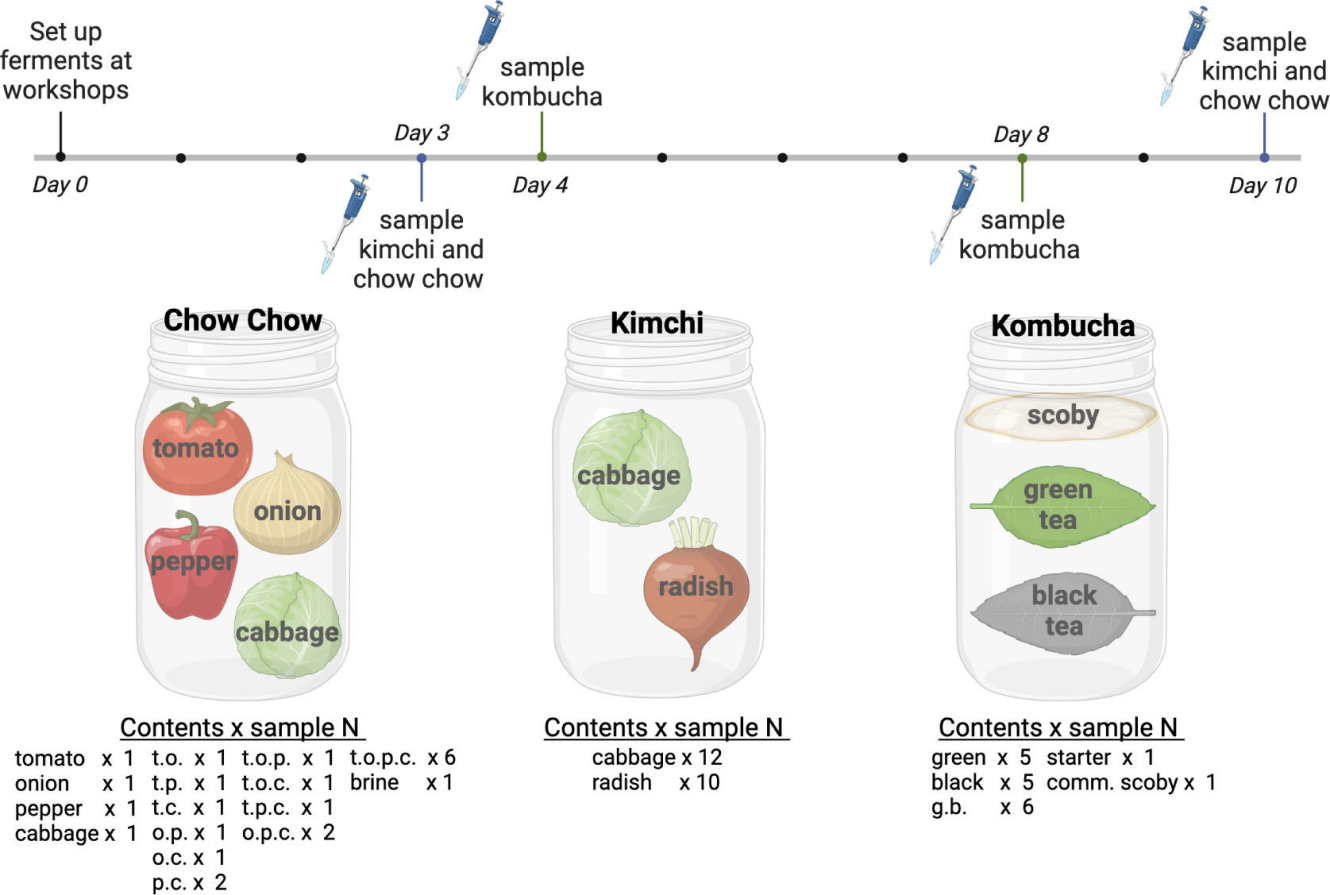
Experimental setup. Fermented food samples and sampling timeline. All ferments were set up on day 0. Liquid from kimchi and chow chow samples was collected for 16S amplicon sequencing, and pH was measured on days 3 and 10. Chow chow pH was additionally measured on day 0, and a brine-only sample from the chow chow experiment was collected on day 3. Aliquots were collected from kombucha samples for 16S and internal transcribed spacer (ITS) amplicon sequencing, and pH was measured on days 0, 4, 8, and 21. Starter liquid samples were also collected on days 4 and 8, and a sample from the commercial SCOBY biofilm was also collected. A list of sample contents and *N*s is found at the bottom of the figure and in Table S1. Created in https://BioRender.com.

For this study, scientists partnered with local fermenting experts to host three participatory science workshops at the NC Museum of Natural Sciences (Raleigh, NC; Fig. 1). Each workshop focused on a specific fermented food: kimchi (a traditional Korean dish of fermented cabbage, radish, and spices), chow chow (a relish fermented in the southern USA containing cabbage, green tomatoes, onion, and green pepper), or kombucha (tea fermented by a symbiotic culture of bacteria and yeast, or SCOBY). Cooking classes were designed with the different expert chefs based on their best practices for teaching workshops. Each workshop began with interactive presentations by the chefs on the history and cultural significance of the food, including samples of the focal ferment. Next, scientists explained which microbes are processing the ferment, which metabolic pathways microbes use to ferment tea or produce substrates, and how the microbial metabolites increase the shelf life and nutritional value of fermented foods. Scientists also explained what metabolic functions the microbes perform (e.g., acid production), the consequences (sour flavor, increased nutritional value, inhibition of colonization by microbes associated with food spoilage, ultimately increasing shelf life), and how the foods would be analyzed in our “science of fermentation” presentations. Scientists then introduced the ecological concepts we were testing in each experiment, and the chefs led participants through the recipes to set up each fermented food experiment (see Supplemental Materials for full recipes). Briefly, to make kimchi, participants mixed pre-salted nappa cabbage or radish with spices and packed the mixture into a jar. To make chow chow, participants packed one to four produce substrates into a jar, then poured enough brine (made with 2% salt and non-chlorinated water) into the jar to cover the substrates. To make kombucha, participants mixed 1/2 cup of sugar with six cups of freshly brewed and cooled black and/or green tea, then added 1/2 cup liquid starter (mature kombucha) and a small piece of the SCOBY biofilm from the previous ferment to the new jar. For kombucha, three starters were used, black tea, green tea, and a mix of green and black tea, and added to the respective fermentation jars. We varied the number of plant substrates in chow chow (1–4 vegetables) and kombucha (1–2 teas) recipes to test the hypothesis that alpha diversity in ferment microbial communities increases with additional substrates. To test the hypothesis that more phylogenetically diverse plant substrates increase the beta diversity in ferment microbial communities, we compared kimchi made from cabbage or radish and kombucha made from green or black teas. We measured changes in diversity and pH over time in all three ferments to test the hypothesis that diversity increases during succession as pH decreases. The numbers of substrate combinations in each group (see Fig. 2; Table S1) were chosen to provide conditions to test for each hypothesis while considering the constraints of workshop class size. The participants followed the recipe and instructions to make their own jars of ferment to enjoy at home, as well as experimental jars that remained in the laboratory on display behind a window for the public to view the fermentation process.

### Sample collection and processing

From chow chow jars, brine (but no plant material) was collected on days 0, 3, and 10 after the experimental setup during the workshop. One brine-only sample, from a jar with brine and no vegetables, was collected on day 3. Kimchi, which creates its own liquid culture, was sampled on days 3 and 10. Liquid culture was collected from the experimental kombucha jars on days 0, 4, 8, and 21. The time points were chosen for pH (all time points) and microbiome samples (kombucha: days 4 and 8; chow chow and kimchi: days 3 and 10) to equate to the same level of acidity with the bacteria associated with the fermentation at that time point. Previous reports of fermentation have shown indicator curves of acidity (i.e., lower pH), and lactic acid bacteria (LAB) were plated on media and species were identified (14). Thus, collections were made in the middle of active fermentation and after fermentation likely reached a plateau. We also sampled a liquid culture of mature kombucha, which was fermented from a blend of green and black tea for 43 days prior to the workshop, at days 4 and 8 of the experiment for comparison to our experimental kombucha communities (Southern Peak Brewery, North Carolina, USA). Kombucha SCOBY biofilm was collected from the commercial brew by cutting a 1/2 cm × 1/2 cm × 1/2 cm cube piece into a 2 mL microfuge tube and using a sterile disposable plastic pellet pestle (cat. no. 12-141-363, Fisherbrand, Fisher Scientific, Pennsylvania, USA) to break up the solid mass.

Two liquid samples were collected from each jar on each sample day. One 1.8 mL sample of liquid was pipetted into a 2 mL sterile microcentrifuge tube included in the PowerFood DNA isolation kit (Qiagen, Hilden, Germany, reference no. 21000-100). We sequenced day 3 and 10 samples from kimchi and chow chow jars, and day 4 and 8 samples from kombucha jars. The second liquid sample from each jar was pipetted into a 2 mL sterile microfuge tube (cat. no. 14-666-313, Fisherbrand, Fisher Scientific, Pennsylvania, USA) for pH testing (Orion 3 STAR, Thermo Scientific, USA). See Fig. 2 and Table S1 for a summary of experimental design including recipes, sampling, and sample sizes.

### DNA extraction and 16S and ITS amplicon sequence libraries

Microbial DNA was isolated from samples as described above. One negative control from each kit was collected by using only buffers (no food sample) and processed using the standard protocol. DNA samples from all three experiments (totaling 123), one chow chow brine-only sample, and the kit controls were stored at −20°C and thawed for DNA amplification using standard PCR methods and 16S rRNA primers with 515FAdapt (5′ TCGTCGGCAGCGTCAGATGTGTATAAGAGACAGGTGCCAGCMGCCGCGGTAA 3′) and 806RmodAdapt (5′ GTCTCGTGGGCTCGGAGATGTGTATAAGAGACAGGGACTACNVGGGTWTCTAAT 3′) primers for Illumina sequencing. The PCR reactions were amplified within a PCR workstation laminar flow hood to reduce contamination, using DreamTaq MasterMix reagent (Thermo Fisher Scientific, Waltham, MA, USA) in 50 µL total reaction volume (DNA template 5 µL, sterile MilliQ water 18 µL, DreamTaq Master Mix 25 µL, each 10 µM primer 1 µL), on an Eppendorf thermal cycler (Eppendorf, Germany) with the following reaction settings: 95°C 3 minutes; 35 cycles of 95°C 30 seconds, 53°C 60 seconds, 72°C 60 seconds; 72°C 10 minutes, then held at 4°C. All samples from the tea experiment (34 liquid, 1 SCOBY, and 2 blank kit controls) were amplified using internal transcribed spacer (ITS; intertransgenic region of rRNA genes) ITS1FAdapt (5′ TCGTCGGCAGCGTCAGATGTGTATAAGAGACAGCTTGGTCATTTAGAGGAAGTAA 3′) and ITS2RAdapt (5′ GTCTCGTGGGCTCGGAGATGTGTATAAGAGACAGGCTGCGTTCTTCATCGATGC 3′) primers, using the same reaction conditions. PCRs were verified by gel electrophoresis for bands at 292 base pairs (bp) for 16S and between 250 and 600 bp for ITS. These products were treated with 2 µL ExoSAP-IT reagent (Affymetrix/USB, Santa Clara, CA, USA) to clean up 23 µL PCR reactions and remove free primers and remaining deoxyribonucleoside triphosphates (dNTPs).

Amplified products were indexed using dual Nextera indexing primers N701-N715 and S502-S503, S505-S508, S510-S511, S513, S515-S518, S520-S522 (Table S2), synthesized from IDT (Integrated DNA Technologies, Inc., Coralville, IA, USA). Reaction parameters were as follows: Phusion HF MM 15 µL, i7 index primer 2.5 µL, i5 index primer 2.5 µL, DNA template 10 µL for a total reaction volume of 30 µL. Reaction settings were as follows: 98°C for 2 minutes; 10 cycles of 98°C for 10 seconds, 53°C for 30 seconds, 72°C for 15 seconds; 72°C for 5 minutes, 4°C pause. Amplified indexed products were cleaned by AMPure XP bead reagent (cat. no. A63881, Beckman Coulter, IN, USA) and resuspended in 21 µL of resuspension (Tris-HCl) buffer. DNA was quantified by Qubit 4 Fluorometer and HS dsDNA Assay (cat. no. Q32854, Thermo Fisher Scientific, Waltham, MA, USA), and pooled to equal molar amounts. The pool was quantified on a Tapestation Analyzer (Agilent, CA, USA) and with qPCR Kapa Quant kit (Roche Sequencing & Life Science, Pleasanton, CA). Illumina MiSeq V3 600 cycle kit used the 4 nM pooled DNA added as input into sequence setup protocol in a final concentration of 10 pM denatured library with 18% PhiX (10 pM) (Illumina, CA, USA).

### Bioinformatic analysis

Raw sequences in FASTQ file format containing the FASTA sequences with quality scores and pre-trimmed adapters and indexes were downloaded from the Illumina BaseSpace repository (Illumina, CA, USA). Sequenced 16S reads were pre-processed in the Qiime2 pipeline (q2) utilizing the DADA2 method to merge paired-end reads (forward reads truncated at 260 bp, reverse reads truncated at 208 bp, and forward reads trimmed 19 bp from the left end, and reverse reads trimmed 20 bp from the left end to remove primer sequences) and to filter for chimeras (via q2‐dada2) (15, 16). Data tables with amplicon sequence variants (ASVs) and representative sequences were generated and exported via biom file from Qiime2 (version 2020.6) (15, 17).

Due to a faint PCR product shown in the first kit control, a statistical method was used to eliminate those reads from the ASV table. A data table of 25 samples from the kimchi experiment as representative ASVs and the extraction kit control ASVs was run through the Decontam R script using the prevalence method (18), and 13 ASVs were eliminated as background contaminants with significant kit control ASV read counts overlapping sample ASV read counts. The resulting ASV data table with contaminant ASVs removed was reimported into Qiime2. The ASV data tables from all three experiments were merged into one table containing 128 samples using q2 feature-table merge. Representative 16S sequences were identified from the merged ASV table using q2 feature-table filter-seqs. All files were exported from Qiime2 for statistical analysis in R (19).

Taxonomic identity of ASVs was determined using the naive Bayesian classifier implemented by the “assignTaxonomy” function in DADA2 R package and the non-redundant Silva taxonomic training database version 138 (release date 2 November 2020) (16, 20, 21). Species identity was assigned using the same database, with exact matching by the “addSpecies” function in DADA2. ASVs assigned to Eukaryota, Chloroplast, or Mitochondria were filtered and removed, along with any reads unassigned at the phylum level. All ASVs classified in the genus *Lactobacillus* were reclassified with the “lactotax” tool (http://lactobacillus.uantwerpen.be/) to follow the emended description of the genus (22). *Lactobacillus* ASVs that were unclassified at the species level by the naive Bayes classifier were searched using BLAST (https://blast.ncbi.nlm.nih.gov/) against the 16S ribosomal RNA sequences database (BioProjects 33175 and 33317; accessed on 9 November 2023). ASVs that met a ≥99% identity threshold for a single genus were assigned to that genus, whereas ASVs that did not meet this criterion were assigned only to the family level.

Sequenced ITS reads from 34 kombucha samples were pre-processed in the Qiime2 pipeline (q2). The q2 cut-adapt trim-paired method was applied using the forward and reverse primer sequences and anchors (^) at the start of the primer sequence and including the linked adapter (reverse-complement of read 1 (forward reads) and the reverse-complement of read 2 (reverse reads)) to accommodate amplicon reads shorter than 250 bp (23). The parameter p-minimum-length 50 was applied to this command to filter out any reads remaining less than 50 bp. The q2 DADA2 method (16) was utilized to merge paired-end reads and filter chimeras. ASV counts and representative sequences were generated, including 16 ASVs, and exported from Qiime2 via biom file for statistical analyses in R. ITS ASV taxonomy was assigned using the naive Bayesian classifier as described above with the UNITE database version 04.02.2020 (release date 4 February 2020) (24).

### Statistical analysis

All statistical analyses were performed in R (19). For alpha diversity metrics and analyses, samples were rarefied to the minimum library size in each respective experiment (see Fig. S1). Rarefaction was only used for the calculation of alpha diversity, because although it can account for impacts of sample size on alpha diversity (25, 26), it may inhibit other analyses (27). Paired Wilcoxon rank-sum tests were used to compare observed richness and Shannon indices between the 1st and 2nd days of sample collection, with Benjamini-Hochberg *P*-value correction for testing multiple alpha diversity measures. For beta diversity analyses, ASV read counts were normalized to proportions of sample library size (relative abundance). Beta diversity was calculated using Bray-Curtis distance, which takes both richness and relative abundance into account. Non-metric multidimensional scaling (NMDS) was used to ordinate Bray-Curtis distances. Permutational multivariate analysis of variance (PERMANOVA) tests were used to assess whether Bray-Curtis distances varied by sample collection day in each experiment. A Wilcoxon rank-sum test was used to compare pH in kimchi samples, as two measurements were taken. Kruskal-Wallis tests were used to compare pH in chow chow and kombucha samples as three and four pH measurements were taken, respectively. Dunn’s *post hoc* test was used for paired comparisons in Kruskal-Wallis tests with Benjamini-Hochberg *P*-value correction for multiple comparisons.

When comparing the impact of the number or type of substrates, only samples from the 2nd day of measurement were compared, as using both time points would falsely inflate the *N* by counting the same samples twice. Only food samples were included in statistical tests and not brine or starters, as there was only one of these types of samples in their respective experiments. Wilcoxon rank-sum tests were used to compare observed richness and Shannon indices by number of substrates (for chow chow and kombucha) or substrate type (for kimchi and kombucha), with Benjamini-Hochberg *P*-value correction for testing multiple alpha diversity measures where appropriate. Kruskal-Wallis tests with Dunn’s *post hoc* with Benjamini-Hochberg *P*-value correction for multiple comparisons were used when there were more than two categories. NMDS ordination was performed on Bray-Curtis distances to observe differences in kimchi and kombucha by substrate type. PERMANOVA tests were performed to assess differences in Bray-Curtis distances by substrate. Number of plants was originally included in the model for kombucha, but was removed as the coefficient was not significant.

An indicator analysis was performed to determine which ASVs were indicators for variables found significant by PERMANOVA. This was performed using the indicspecies package (28) and included ASVs present in ≥10% of samples and a mean relative abundance ≥0.1% in its respective experiment. ASVs were considered to be indicators if the false-discovery rate corrected *P*-value (*q*-value) was ≤0.05.

## RESULTS

### Bacterial and fungal communities

**Fig 3:**
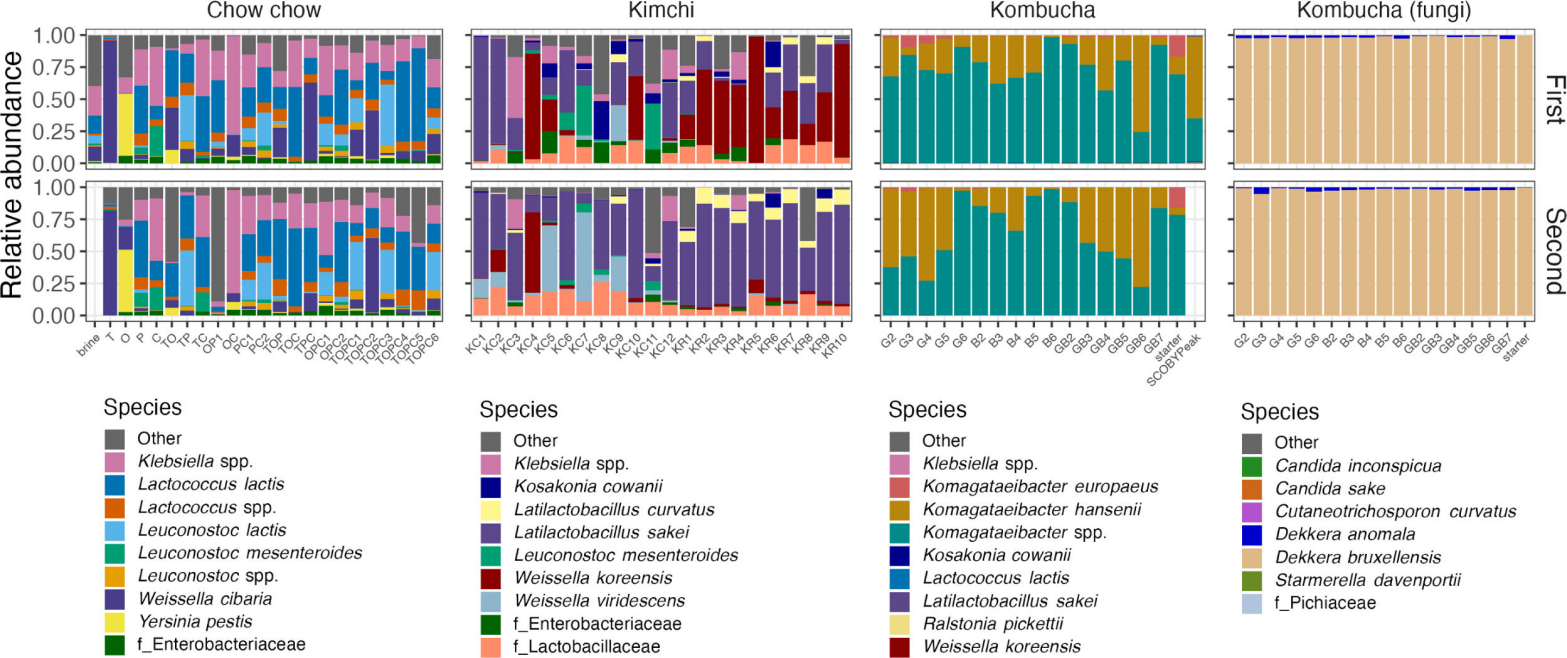
Sample microbial communities. Stacked bar plots depicting relative abundance of the top 9 bacterial or 7 most abundant fungal species in each sample in each experiment. Taxa that were not resolved to the species level are noted. Sample names on the *x*-axis reflect the first letter of the plant substrate(s) or the sample type such as brine, starter, or SCOBY.

Bacterial communities were measured using amplicon sequencing of the 16S rRNA gene of samples collected on days 3 and 10 for chow chow and kimchi samples, or on days 4 and 8 for kombucha samples. Figure 3 depicts the top 9 most abundant bacterial species in each experiment and, also, in the right-most panels, the top 7 fungal species for kombucha samples. Bacterial communities measured on day 3 or 4 are shown as the 1st day, and communities measured on day 8 or 10 are shown as the 2nd day. Fungal communities were measured using amplicon sequencing of the ITS region in kombucha samples from days 4 and 8. Overall, the most abundant species within each type of ferment were consistent among samples. Some abundant species appeared in multiple ferments, including *Leuconostoc mesenteroides*, *Lactococcus lactis*, *Latilactobacillus sakei*, and *Weissella koreensis*. No clear patterns or differences were observed in chow chow samples, except that the tomato-based chow chow was dominated by *Weissella cibaria* at both time points, whereas other recipes demonstrated more even abundances of species. On day 3, the radish-based kimchi was abundant in *Weissella koreensis*, but this species was in low abundance on day 10, when these samples were dominated by *Latilactobacillus sakei. Leuconostoc mesenteroides* was present in high abundance in some day 3 cabbage kimchi samples. Kombucha bacterial communities were dominated by *Komagataeibacter* at both time points (mean = 99.8%), and *Dekkera bruxellensis* represented a mean of 98% of fungal communities.

### Diversity of ferment microbial communities did not increase with added substrates

**Fig 4:**
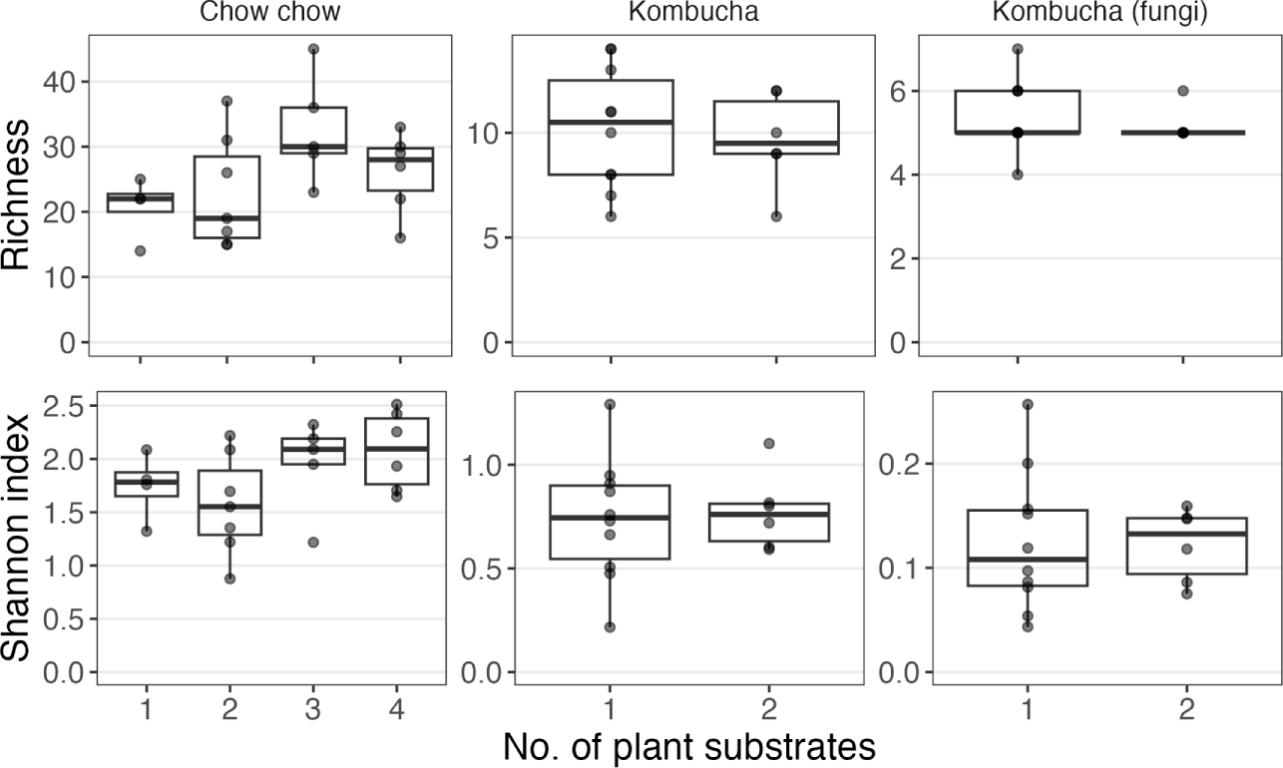
Impacts of substrate number on diversity. Alpha diversity, measured as observed richness and Shannon index, by the number of plant species in each sample. Samples were rarefied to the minimum library size in each respective experiment (see Fig. S1), and only samples from the second time point were assessed. No significant differences were found among the observed richness or Shannon indices in chow chow samples (by Kruskal-Wallis test) or the tea bacteria or fungi samples (by Wilcoxon rank sum). Note that axes differ among panels, as richness and Shannon index were much lower in kombucha than in chow chow.

To assess the hypothesis that a greater diversity of resources would favor greater diversity of consumers, we compared the alpha diversity of microbial communities in chow chow with one to four vegetable substrates and kombucha with one or two teas. For this analysis, we compared mature communities from the second time point (day 10). Failing to support our hypothesis, alpha diversity, measured by ASV richness and Shannon index, was not significantly impacted by the number of substrates in chow chow or kombucha (*P* > 0.05, Fig. 4).

### Phylogenetically diverse substrates support distinct ferment microbial communities

**Fig 5:**
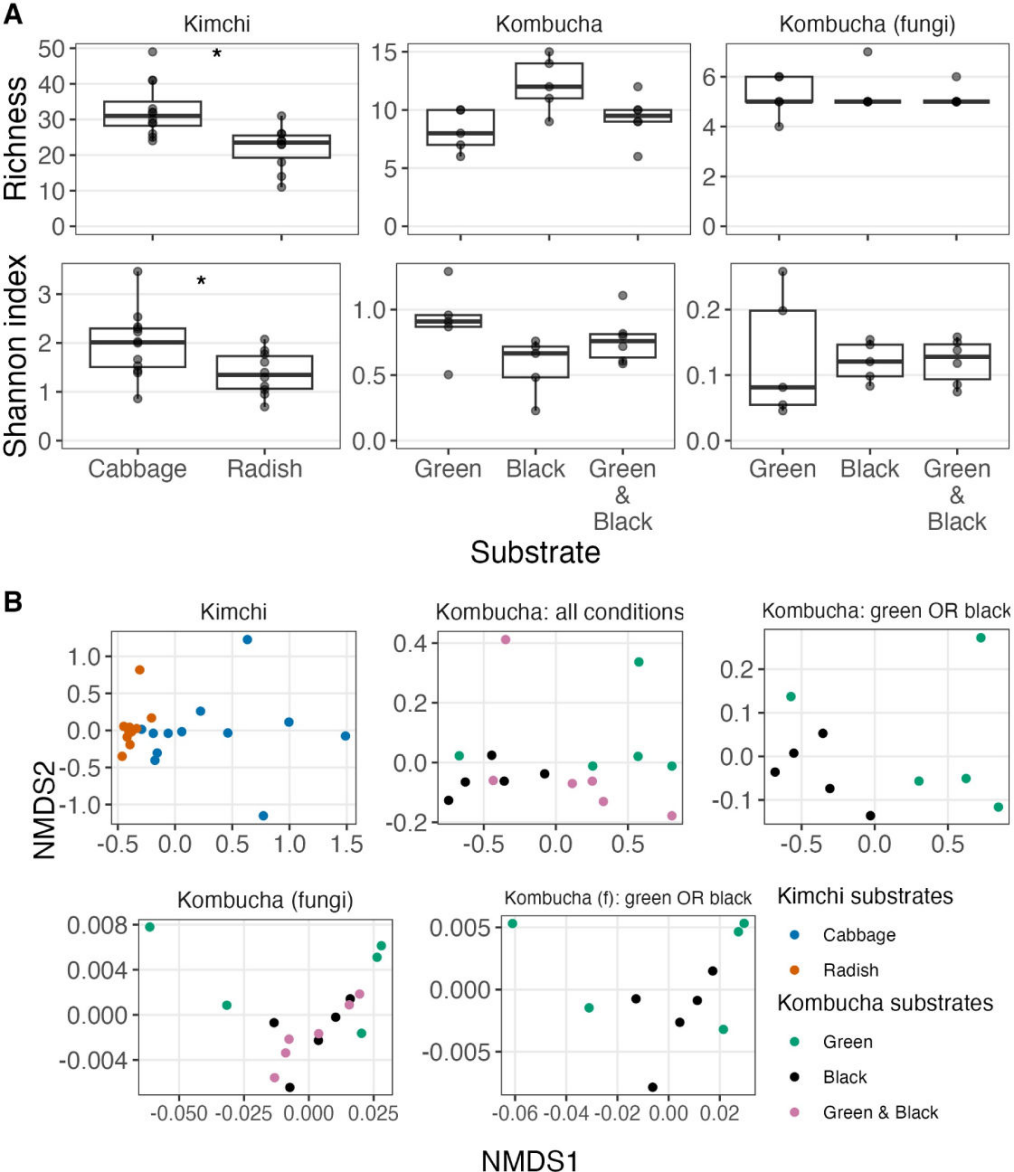
Impacts of substrate type. (**A**) Alpha diversity, measured as richness and Shannon index, by plant ingredients used. Samples were rarefied to the minimum library size in each respective experiment (see Fig. S1), and only samples from the second time point were assessed. The effect of plant ingredients on observed richness and Shannon index in kimchi samples was assessed with Wilcoxon rank-sum tests. Alpha diversity was greater in cabbage samples using observed richness (*P* = 0.005) and Shannon diversity (*P* = 0.025). Significant differences in alpha diversity by observed richness and Shannon indices were not demonstrated by Kruskal-Wallis tests in kombucha bacteria or fungi. Note that axes differ among panels. (**B**) Non-metric multidimensional scaling on Bray-Curtis distances of ASV relative abundances. Samples were not rarefied before analysis. PERMANOVA tests were used to measure the impact of substrates on communities. Kimchi communities differed significantly based on substrate (*P* = 0.001). When comparing single-substrate kombucha communities, bacterial communities differed significantly (*P* = 0.042) but fungal communities did not (*P* = 0.772). When comparing all three kombucha substrate groups, there were differences among bacterial communities (*P* = 0.05) but not fungal communities (*P* = 0.93).

We next assessed whether the composition of microbial communities would differ based on substrates that were not closely phylogenetically related (cabbage or radish kimchi) compared to substrates that were closely related (green or black tea kombucha; Fig. 5). We again used samples from the second time point for this analysis: day 8 for kombucha and day 10 for kimchi samples. Consistent with our hypothesis, alpha and beta diversity varied between cabbage and radish kimchi samples. Kimchi made from cabbage demonstrated significantly greater ASV richness (Wilcoxon rank sum, *P* = 0.005) and Shannon diversity (*P* = 0.025). NMDS ordination on Bray-Curtis distances revealed statistically distinct microbial communities associated with radish versus cabbage kimchi samples (PERMANOVA, *P* = 0.001). Alpha diversity metrics did not vary significantly among the similar tea substrates (Kruskal-Wallis, *P* > 0.05). However, the tea substrates yielded some differences in beta diversity. Bray-Curtis distances of bacterial communities between kombucha samples made only with green or black tea differed significantly (PERMANOVA, *P* = 0.042), though fungal communities did not (*P* = 0.772). Differences in Bray-Curtis distances were found among all three kombucha recipes (including a mixture of green and black teas) in bacterial communities (*P* = 0.05) but not fungal communities (*P* = 0.93).

We next performed indicator analyses for comparisons where the above PERMANOVA tests were significant in order to determine whether the substrates selected for specific microbial taxa. In this analysis, we only included ASVs that were detected in ≥10% of samples, and we considered ASVs to be indicators if the false-discovery rate corrected *P*-value (*q*-value) was ≤0.05. Eleven ASVs were found to be indicators for cabbage in kimchi, and two were indicators of radish as a substrate. The mean relative abundance (as a percent) for each plant substrate is presented in Table 1, and the relative abundances of these ASVs in each sample are presented in Fig. S2. Most of the indicator ASVs were present at low abundance (less than 10%) in all samples, except for *Leuconostoc mesenteroides* and one of three ASVs classified as *Weissella viridescens*. No ASVs were indicators of tea substrate, suggesting that the two tea types, which were the same species, did not select for specific microbial taxa.

**TABLE 1:** Significant indicator ASVs in kimchi^*a*^

No.	Family	Genus	Species	Stat	*P*-value	*q*-value	Mean cabbage abund. (%)	Mean radish abund. (%)
Cabbage								
1	Acetobacteraceae	*Komagataeibacter*		0.938	0.001	0.003	0.54	0.08
2	Lactobacillaceae			0.866	0.007	0.015	0.54	0.04
3	Lactobacillaceae			0.901	0.002	0.006	0.94	0.08
4	Lactobacillaceae			0.911	0.001	0.003	1.75	0.11
5	Lactobacillaceae			0.969	0.001	0.003	2.70	0.13
6	Leuconostocaceae	*Leuconostoc*	*mesenteroides*	0.980	0.001	0.003	4.88	0.07
7	Leuconostocaceae	*Leuconostoc*		0.878	0.007	0.015	1.08	0.04
8	Leuconostocaceae	*Weissella*	*viridescens*	0.816	0.006	0.015	0.61	0.00
9	Leuconostocaceae	*Weissella*	*viridescens*	0.993	0.001	0.003	5.30	0.07
10	Leuconostocaceae	*Weissella*	*viridescens*	0.975	0.001	0.003	2.64	0.13
11	Streptococcaceae	*Lactococcus*	*lactis*	0.971	0.026	0.048	0.85	0.07
Radish								
12	Erwiniaceae	*Pantoea*	*vagans*	0.906	0.019	0.038	0.68	1.47
13	Lactobacillaceae	*Latilactobacillus*	*curvatus*	0.977	0.001	0.003	0.49	6.20

^*a*^ Significant indicator ASVs and the values for statistic, *P*-value, *q*-value, mean abundance in percentage for each vegetable ingredient.

### pH decreased and community composition changed during succession

Liquid culture pH was measured on days 0, 3, and 10 for chow chow, days 3 and 10 for kimchi, and days 0, 4, 8, and 21 for kombucha. As expected, pH decreased over time as fermentation progressed (Fig. S3A), likely due to the growth of lactic acid and acetic acid-producing bacteria. We also assessed the change in bacterial communities over time for all fermentations and fungal communities for kombucha, using alpha and beta diversity to test our hypothesis that richness and diversity would increase during succession. Observed richness and Shannon indices did not change between time points in any fermentation (Fig. S3B, Wilcoxon rank-sum, *P* > 0.05). PERMANOVA tests on Bray-Curtis distances suggested that community composition changed over time in chow chow (*P* = 0.007) and kimchi samples (*P* = 0.009), but not kombucha bacteria (*P* = 0.963) or fungi (*P* = 0.689). Ordinations by NMDS on Bray-Curtis distances showed that kimchi samples were more varied on day 3 but more similar on day 10 (Fig. S3C). The microbial communities of the mature kombucha starter were more similar to each other than to experimental samples at both time points, suggesting that communities stabilized with constrained membership after day 21, likely in response to increasing acidity. Kombucha samples clustered separately from the commercial SCOBY biofilm (Fig. S3C).

Indicator analyses also were performed to test for indicator taxa associated with days elapsed in the chow chow and kimchi samples, based on significant coefficients found in the above PERMANOVA analyses. No ASVs were found to be indicators of sampling day.

## DISCUSSION

Our team composed of evolutionary biologists, ecologists, microbiologists, chefs, and educators used the fermented foods of chow chow, kimchi, and kombucha to study basic microbial ecology questions in a system conducive to public engagement. Fermented food experiments were set up in workshop-style classes, and microbial communities were measured with amplicon sequencing. The microbiomes detected in each food experiment varied, but species in the order Lactobacillales were among the most abundant in all experiments. Bacteria within this order are known for producing lactic acid, and LAB are necessary for the fermentation of vegetables (29). Traditional culture methods and previous sequencing studies have long shown that *Leuconostoc*, *Lactobacillus*, and *Weissella* are predominant genera in kimchi and other fermented vegetable dishes (30–35), and these were among the most abundant genera in chow chow and kimchi samples. *Leuconostoc mesenteroides* in particular has been associated with early successional stages in vegetable fermentation (30, 32, 36), and was among the most abundant species in some day 3 kimchi and chow chow samples. The LAB in fermented foods are known to decrease the pH, creating an acidic environment; this phenomenon observed in our study has been well documented (5, 34, 35). The increasing similarity among microbial replicate communities of microbes in kimchi between day 3 and day 10 suggests that communities converge in response to increasing acidity. Kombucha was overwhelmingly dominated by acetic acid bacteria in the family Acetobacteraceae, mostly from the genus *Komagataeibacter*, consistent with previous studies (37–39). Fungal kombucha communities were overwhelmingly dominated by *Dekkera bruxellensis*, a species that has been shown to be abundant in other kombucha microbiomes (38).

Within the ferments our participants produced, we tested the impacts of substrate diversity and substrate type on communities. In contrast to results from old fields and microcosms (9–12), a greater diversity of plants (in this case as substrates) did not predictably lead to increases in the alpha diversity of dependent species (in this case, fermentation microbes), whether in chow chow or kombucha. Future studies could increase the maximum number of substrates tested to further investigate a potential impact of substrate number on diversity. However, our results may also reflect differences in the drivers or constraints that may govern alpha diversity dynamics in closed jars of fermented foods, versus larger open environments.

In our kimchi recipes, we also tested the impact of two phylogenetically distant substrates, cabbage and radish, on bacterial communities. Bacterial genera have been shown to vary among these and other kimchi substrates, though variation tends to be driven more by sampling time (early versus late in fermentation) or salt concentration (34). Our study measured bacterial and fungal communities using exact ASVs, which can often identify bacteria at the species or subspecies level. We found that the bacterial communities measured by ASVs in cabbage kimchi differed from those in radish kimchi in both alpha and beta diversity. Alpha diversity was greater in cabbage species, and this was also reflected in the greater number of ASVs and species that were found to be indicators of cabbage as a substrate. Future research could test whether the impact of substrate on variation of microbial species of ferments is fueled by greater diversity of source carbohydrate substrates or environmental microbes in cabbage leaves compared to radish (roots). Future research could also explore microbial membership, succession, and fermentation dynamics associated with ecologically distinct plant materials within the same species, such as leaves versus roots. Furthermore, three cabbage indicator ASVs belonged to the species *Weissella viridescens*, and one of these ASVs appeared at greater concentrations than others (Fig. S2), showing potential within-species variation.

In contrast to our assessments of microbial communities of phylogenetically distinct substrates, the alpha diversity of bacterial and fungal communities in kombucha did not vary based on tea type. Black, green, or a mixture of both teas did not impact alpha diversity of bacteria or fungi. Bacterial communities varied between green and black tea, but not fungal communities, which has been shown previously (38). However, no indicator species were identified, suggesting that the two tea types do not select for specific ASVs or microbial species. Based on NMDS, black tea samples show less variation, or more constrained variation, in both bacteria and fungi compared to green tea. During the production of black tea, oxidation of polyphenols is promoted, and the main compositional difference between green and black tea is the concentration and type of polyphenols (40). Future studies should investigate the impact of nutrient variation within these teas and impacts on microbial diversity. Functional differences between these bacterial communities could also be examined using metatranscriptomics.

Overall, we were able to successfully use fermented foods as model systems to study microbial ecology. Although the ferment microbiomes were similar to previous experiments, future experiments could improve on technical aspects of the methods. To further ensure representative samples of the ferments, future experiments could sample and centrifuge larger volumes of the ferment brines to capture larger abundances of bacteria. Additionally, as the LABs in vegetable ferments live on plant material (41), future experiments could further ensure representative sampling by blending and filtering vegetable ferment samples (33, 34). In addition to our scientific goal of using fermented foods as model systems, the other major goal of this study was to engage with chefs and the public around science and food. These workshops provided a casual space where cooks, scientists, and the public could share and appreciate each other’s expertise around food. Chefs delighted in the opportunity to teach their favorite recipes and answer questions about different recipe parameters, which often reflect familial tradition and/or personal preferences. This study prompted additional scientific questions for Sarah Michalski, who led the kombucha workshops, regarding the impacts of changing environments on kombucha. Such changes include the impact of historical changes to recipes, i.e., the use of sugar instead of honey or using starters from varying geographical locations. Sarah Michalski further reflected that “how we share cultures and how we engage with the public matters, and it happens more than people think—and that represents an untapped opportunity to share how your environment and what you put into your ferment matters” (see the supplemental material for full reflections from some chef and scientist authors). The scientist co-authors were able to collaborate with the chef co-authors to plan the workshop and select vegetable and tea substrates and have noted that chefs’ curiosities and gaps in knowledge can inform hypothesis development and experimental design when studying ferments. Scientists were able to engage as participants during the food preparation, and these recipes provided novel protocols for experimental procedures. Participants asked questions about both the science and the art of food-making, which further enriched conversation and informed the research, and they were able to appreciate the microbes that make it all work. Chefs were able to provide insight to both scientists and participants on the histories and “trade secrets” of these foods. For example, Elizabeth Weichel and Amanda Matson of Piedmont Picnic described how the ingredients and proportions in chow chow vary across individuals, gardens, and seasons, depending on what produce remains to be harvested at the end of the season. We also noted how scientist protocols and chef recipes can differ. For example, Soo Hee “Mama” Kwon embodies the “I don’t know how much, I just add the ‘right amount’” approach to executing internalized recipes that are handed down across generations. It takes time and discussion to articulate automatic actions as a protocol that others can follow.

Although we believe this study was successful in its research goals, we note that the project also included illuminating pitfalls. One challenge was simply orchestration. Simultaneously carrying out an experiment and a cooking class in a confined space required a great deal of coordination. On the one hand, it might have (in retrospect) been useful to provide more ecological context to participants. On the other hand, this project worked because it was brief enough to “fit” into the time and space a cooking class allows. Our lesson, in this context, is that combining a cooking-class approach with the practice of science and science education inevitably requires tradeoffs. It is useful, as a team, to understand how to optimize those tradeoffs given the goals. In future iterations, we can imagine adding additional explanation, such as utilizing data from previous studies to walk participants through the data analysis process that will be used on the samples they generate. Additional experiments, samples, and other features could also be added, but it is clear that we would need to do so with the constraints of the approach in mind. Certainly, in the future, we would add more opportunities for the participants to share their ideas in advance and to share their insights in the aftermath. This could be in the form of formal surveys which have been successfully deployed in fermented foods participatory science research and also used to demonstrate increased interest and knowledge throughout study participation (42). Such surveys or questionnaires could measure participant attitudes toward microbes before versus after attending workshops or other educational events, to assess whether and how effectively education alters perception and value of microbes. Study results and participants’ interpretations could also be shared in a dedicated follow-up event or focus group, and/or via annotated figures, webinars, or websites (see http://robdunnlab.com/projects/sourdough/ for report-back examples from the Global Sourdough Project). Another challenge with a sequencing-based project relates to timing. Providing data back to participants is most exciting for participants when done relatively soon after an event. In practice, the extended time required for sequencing and data processing (though progressively less so) makes immediate report-back difficult. The greater challenge is the time required to analyze data, both formally as well as the contextualization necessary to consider complex data. Our author group has now participated in dozens of sequencing-based participatory science projects. Rarely does this process take less than 2 years (in our case, it took nearly 7). One solution to this delay is regular communication.

This project was highlighted in the window of the Genomics and Microbiology Research Lab at the North Carolina Museum of Natural Sciences, and a research cart was created using preliminary study results and a display of jars of fermented foods. A presentation with discussion was given in the Teen Science Café event to engage teens interested in science. These resources successfully engaged the public at museum events for the past 6 years. It is probable that this Museum Community Engagement model is worthwhile for other projects that may have an extended trajectory for formal publication. Museum events are also likely more successful in disseminating results to the broad public compared to scientific journals. Future outreach efforts should ensure accessibility to wide audiences. Although museums are excellent locations for informal education, they may attract participants who are more affluent or already have some exposure to or interest in science, microbes, and fermentation. Future events could include advertising and hosting at community centers, public schools, farmers markets, college campuses, or lifelong learning centers. Ideally, a workshop series would enable scientists, fermenters, and participants to form long-term relationships and design experiments to study personally and culturally important fermented foods with intergenerational community members.

### Conclusion 

This project demonstrates the utility of fermented foods as model systems for microbial ecology, while successfully collaborating with chefs and participant scientists. We are also aware that this effort is part of a growing portfolio of food science fermentation events that enable the public to participate in the science of food in new ways, with varying levels of participatory scientist contribution. Participatory science engagement can range from participants submitting home-made fermented food samples (3, 43) to attending community events or talks (42), similar to this study. These efforts have included a range of fermented foods including sourdough starters, kefir, and a variety of fermented vegetables. Additional educational programming has showcased a wide variety of science and cooking skills through university lecture series that include online courses, public lectures, and materials for all ages (44). Furthermore, researchers have described broad benefits of participatory science in enhancing public knowledge of science (45). Some researchers have also proposed that food and agriculture participatory science efforts can benefit food security, food safety, flavor innovation, biodiversity, and more (46).

## Data Availability

Analysis code and processed ASV counts with taxonomy and sample metadata are available at https://github.com/hannalberman/ferments_ecology_workshops. Raw sequencing data isare available in the Sequence Read Archive under BioProject PRJNA1214294.

## SUPPLEMENTAL MATERIAL

Supplemental material spectrum.02660-24-s0001.pdf Tables S1 and S2, Fig. S1 to S3, recipes, and reflections.

ASM does not own the copyrights to Supplemental Material that may be linked to, or accessed through, an article. The authors have granted ASM a non-exclusive, world-wide license to publish the Supplemental Material files. Please contact the corresponding author directly for reuse.

## References

[1] Croft GK. 2019. The US land-grant university system: an overview. CRS Report 45897

[2] Kassel K, Martin A. 2024. Ag and food sectors and the economy. USDA Economic Research Service. Available from: https://www.ers.usda.gov/data-products/ag-and-food-statistics-charting-the-essentials/ag-and-food-sectors-and-the-economy. Retrieved 13 Mar 2024.

[3] Landis EA, Oliverio AM, McKenney EA, Nichols LM, Kfoury N, Biango-Daniels M, Shell LK, Madden AA, Shapiro L, Sakunala S, Drake K, Robbat A, Booker M, Dunn RR, Fierer N, Wolfe BE. 2021. The diversity and function of sourdough starter microbiomes. Elife 10:e61644. doi:10.7554/eLife.61644 33496265 PMC7837699

[4] Reese AT, Madden AA, Joossens M, Lacaze G, Dunn RR. 2020. Influences of ingredients and bakers on the bacteria and fungi in sourdough starters and bread. mSphere 5:e00950-19. doi:10.1128/mSphere.00950-19 31941818 PMC6968659

[5] McKenney EA, Nichols LM, Alvarado S, Hardy S, Kemp K, Polmanteer R, Shoemaker A, Dunn RR. 2023. Sourdough starters exhibit similar succession patterns but develop flour-specific climax communities. PeerJ 11:e16163. doi:10.7717/peerj.16163 37810791 PMC10559884

[6] Calvert MD, Madden AA, Nichols LM, Haddad NM, Lahne J, Dunn RR, McKenney EA. 2021. A review of sourdough starters: ecology, practices, and sensory quality with applications for baking and recommendations for future research. PeerJ 9:e11389. doi:10.7717/peerj.11389 34026358 PMC8117929

[7] Gardner MR, Ashby WR. 1970. Connectance of large dynamic (cybernetic) systems: critical values for stability. Nature 228:784. doi:10.1038/228784a0 5472974

[8] May RM. 1973. Stability and complexity in model ecosystems. Princeton University Press. Available from: https://play.google.com/store/books/details?id=53y-DwAAQBAJ 4723571

[9] Gamfeldt L, Hillebrand H, Jonsson PR. 2005. Species richness changes across two trophic levels simultaneously affect prey and consumer biomass. Ecol Lett 8:696–703. doi:10.1111/j.1461-0248.2005.00765.x

[10] Haddad NM, Tilman D, Haarstad J, Ritchie M, Knops JM. 2001. Contrasting effects of plant richness and composition on insect communities: a field experiment. Am Nat 158:17–35. doi:10.1086/320866 18707312

[11] Siemann E, Tilman D, Haarstad J, Ritchie M. 1998. Experimental tests of the dependence of arthropod diversity on plant diversity. Am Nat 152:738–750. doi:10.1086/286204 18811348

[12] Knops JMH, Tilman D, Haddad NM, Naeem S, Mitchell CE, Haarstad J, Ritchie ME, Howe KM, Reich PB, Siemann E, Groth J. 1999. Effects of plant species richness on invasion dynamics, disease outbreaks, insect abundances and diversity. Ecol Lett 2:286–293. doi:10.1046/j.1461-0248.1999.00083.x 33810630

[13] Kumar S, Suleski M, Craig JM, Kasprowicz AE, Sanderford M, Li M, Stecher G, Hedges SB. 2022. Timetree 5: an expanded resource for species divergence times. Mol Biol Evol 39:msac174. doi:10.1093/molbev/msac174 35932227 PMC9400175

[14] Stamer JR, Stoyla BO, Dunckel BA. 1971. Growth rates and fermentation patterns of lactic acid bacteria associated with the sauerkraut fermentation. J Milk Food Technol 34:521–525. doi:10.4315/0022-2747-34.11.521

[15] Bolyen E, Rideout JR, Dillon MR, Bokulich NA, Abnet CC, Al-Ghalith GA, Alexander H, Alm EJ, Arumugam M, Asnicar F, et al.. 2019. Reproducible, interactive, scalable and extensible microbiome data science using QIIME 2. Nat Biotechnol 37:852–857. doi:10.1038/s41587-019-0209-9 31341288 PMC7015180

[16] Callahan BJ, McMurdie PJ, Rosen MJ, Han AW, Johnson AJA, Holmes SP. 2016. DADA2: high-resolution sample inference from Illumina amplicon data. Nat Methods 13:581–583. doi:10.1038/nmeth.3869 27214047 PMC4927377

[17] McDonald D, Clemente JC, Kuczynski J, Rideout JR, Stombaugh J, Wendel D, Wilke A, Huse S, Hufnagle J, Meyer F, Knight R, Caporaso JG. 2012. The biological observation matrix (BIOM) format or: how I learned to stop worrying and love the ome-ome. Gigascience 1:7. doi:10.1186/2047-217X-1-7 23587224 PMC3626512

[18] Davis NM, Proctor DM, Holmes SP, Relman DA, Callahan BJ. 2018. Simple statistical identification and removal of contaminant sequences in marker-gene and metagenomics data. Microbiome 6:226. doi:10.1186/s40168-018-0605-2 30558668 PMC6298009

[19] R Core Team. 2021. R: a language and environment for statistical computing. R Foundation for Statistical Computing, Vienna, Austria.

[20] Yilmaz P, Parfrey LW, Yarza P, Gerken J, Pruesse E, Quast C, Schweer T, Peplies J, Ludwig W, Glöckner FO. 2014. The SILVA and “All-species living tree project (LTP)” taxonomic frameworks. Nucleic Acids Res 42:D643–D648. doi:10.1093/nar/gkt1209 24293649 PMC3965112

[21] Quast C, Pruesse E, Yilmaz P, Gerken J, Schweer T, Yarza P, Peplies J, Glöckner FO. 2013. The SILVA ribosomal RNA gene database project: improved data processing and web-based tools. Nucleic Acids Res 41:D590–D596. doi:10.1093/nar/gks1219 23193283 PMC3531112

[22] Zheng J, Wittouck S, Salvetti E, Franz C, Harris HMB, Mattarelli P, O’Toole PW, Pot B, Vandamme P, Walter J, Watanabe K, Wuyts S, Felis GE, Gänzle MG, Lebeer S. 2020. A taxonomic note on the genus Lactobacillus: description of 23 novel genera, emended description of the genus Lactobacillus Beijerinck 1901, and union of Lactobacillaceae and Leuconostocaceae. Int J Syst Evol Microbiol 70:2782–2858. doi:10.1099/ijsem.0.004107 32293557

[23] Martin M. 2011. Cutadapt removes adapter sequences from high-throughput sequencing reads. EMBnet j 17:10. doi:10.14806/ej.17.1.200

[24] Abarenkov K, Zirk A, Piirmann T, Pöhönen R, Ivanov F, Nilsson RH, Kõljalg U. 2020. UNITE general FASTA release for Fungi Version 04.20.2020

[25] Sanders HL. 1968. Marine benthic diversity: a comparative study. Am Nat 102:243–282. doi:10.1086/282541

[26] Willis AD. 2019. Rarefaction, alpha diversity, and statistics. Front Microbiol 10:2407. doi:10.3389/fmicb.2019.02407 31708888 PMC6819366

[27] McMurdie PJ, Holmes S. 2014. Waste not, want not: why rarefying microbiome data is inadmissible. PLoS Comput Biol 10:e1003531. doi:10.1371/journal.pcbi.1003531 24699258 PMC3974642

[28] De Caceres M, Jansen F, De Caceres MM. 2016. Package “indicspecies.” indicators 8

[29] Di Cagno R, Coda R, De Angelis M, Gobbetti M. 2013. Exploitation of vegetables and fruits through lactic acid fermentation. Food Microbiol 33:1–10. doi:10.1016/j.fm.2012.09.003 23122495

[30] Plengvidhya V, Breidt F Jr, Lu Z, Fleming HP. 2007. DNA fingerprinting of lactic acid bacteria in sauerkraut fermentations. Appl Environ Microbiol 73:7697–7702. doi:10.1128/AEM.01342-07 17921264 PMC2168044

[31] Kim M, Chun J. 2005. Bacterial community structure in kimchi, a Korean fermented vegetable food, as revealed by 16S rRNA gene analysis. Int J Food Microbiol 103:91–96. doi:10.1016/j.ijfoodmicro.2004.11.030 16084269

[32] Xiong T, Guan Q, Song S, Hao M, Xie M. 2012. Dynamic changes of lactic acid bacteria flora during Chinese sauerkraut fermentation. Food Control 26:178–181. doi:10.1016/j.foodcont.2012.01.027

[33] Nguyen DTL, Van Hoorde K, Cnockaert M, De Brandt E, Aerts M, Binh Thanh L, Vandamme P. 2013. A description of the lactic acid bacteria microbiota associated with the production of traditional fermented vegetables in Vietnam. Int J Food Microbiol 163:19–27. doi:10.1016/j.ijfoodmicro.2013.01.024 23500611

[34] Lee M, Song JH, Jung MY, Lee SH, Chang JY. 2017. Large-scale targeted metagenomics analysis of bacterial ecological changes in 88 kimchi samples during fermentation. Food Microbiol 66:173–183. doi:10.1016/j.fm.2017.05.002 28576366

[35] Jung JY, Lee SH, Kim JM, Park MS, Bae J-W, Hahn Y, Madsen EL, Jeon CO. 2011. Metagenomic analysis of kimchi, a traditional Korean fermented food. Appl Environ Microbiol 77:2264–2274. doi:10.1128/AEM.02157-10 21317261 PMC3067442

[36] Jung JY, Lee SH, Jin HM, Hahn Y, Madsen EL, Jeon CO. 2013. Metatranscriptomic analysis of lactic acid bacterial gene expression during kimchi fermentation. Int J Food Microbiol 163:171–179. doi:10.1016/j.ijfoodmicro.2013.02.022 23558201

[37] Marsh AJ, O’Sullivan O, Hill C, Ross RP, Cotter PD. 2014. Sequence-based analysis of the bacterial and fungal compositions of multiple kombucha (tea fungus) samples. Food Microbiol 38:171–178. doi:10.1016/j.fm.2013.09.003 24290641

[38] Coton M, Pawtowski A, Taminiau B, Burgaud G, Deniel F, Coulloumme-Labarthe L, Fall A, Daube G, Coton E. 2017. Unraveling microbial ecology of industrial-scale Kombucha fermentations by metabarcoding and culture-based methods. FEMS Microbiol Ecol 93. doi:10.1093/femsec/fix048 28430940

[39] Villarreal-Soto SA, Bouajila J, Pace M, Leech J, Cotter PD, Souchard J-P, Taillandier P, Beaufort S. 2020. Metabolome-microbiome signatures in the fermented beverage, kombucha. Int J Food Microbiol 333:108778. doi:10.1016/j.ijfoodmicro.2020.108778 32731153

[40] Graham HN. 1992. Green tea composition, consumption, and polyphenol chemistry. Prev Med 21:334–350. doi:10.1016/0091-7435(92)90041-f 1614995

[41] Louw NL, Lele K, Ye R, Edwards CB, Wolfe BE. 2023. Microbiome assembly in fermented foods. Annu Rev Microbiol 77:381–402. doi:10.1146/annurev-micro-032521-041956 37713453

[42] Walsh LH, Breselge S, Martin JGP, Coakley M, Ferguson E, Stapleton A, Crispie F, O’Toole PW, Cotter PD. 2024. Kefir4All, a citizen science initiative to raise awareness of the roles that microbes play in food fermentation. J Microbiol Biol Educ 25:e0015523. doi:10.1128/jmbe.00155-23 38661415 PMC11044645

[43] Thierry A, Madec M-N, Chuat V, Bage A-S, Picard O, Grondin C, Rué O, Mariadassou M, Marché L, Valence F. 2023. Microbial communities of a variety of 75 homemade fermented vegetables. Front Microbiol 14:1323424. doi:10.3389/fmicb.2023.1323424 38163080 PMC10757351

[44] Sörensen PM, Mouritsen OG. 2019. Science education and public understanding of science via food, cooking, and flavour. Int J Gastron Food Sci 15:36–47. doi:10.1016/j.ijgfs.2018.11.006

[45] Bonney R, Phillips TB, Ballard HL, Enck JW. 2016. Can citizen science enhance public understanding of science? Public Underst Sci 25:2–16. doi:10.1177/0963662515607406 26445860

[46] Ryan SF, Adamson NL, Aktipis A, Andersen LK, Austin R, Barnes L, Beasley MR, Bedell KD, Briggs S, Chapman B, et al.. 2018. The role of citizen science in addressing grand challenges in food and agriculture research. Proc Biol Sci 285:20181977. doi:10.1098/rspb.2018.1977 30464064 PMC6253361

