# Supplementary material for: Cooking-class style fermentation as a context for co-created science and engagement
Source: Microbiol Spectr. 2025 Aug 15;13(10):e02660-24. doi: 10.1128/spectrum.02660-24 (PMC12502524; doi:10.1128/spectrum.02660-24)
Supplement: Supplemental material — Tables S1 and S2, Fig. S1 to S3, recipes, and reflections. [file spectrum.02660-24-s0001.pdf]

Supplemental Tables

Table S1. Sample contents

Contents	N samples
**Chow chow**	
tomato	1
onion	1
pepper	1
cabbage	1
tomato & onion	1
tomato & pepper	1
tomato & cabbage	1
onion & pepper	1
onion & cabbage	1
pepper & cabbage	2
tomato, onion & pepper	1
tomato, onion & cabbage	1
tomato, pepper & cabbage	1
onion, pepper & cabbage	2
tomato, onion, pepper & cabbage	6
brine	1
**Kimchi**	
cabbage	12
radish	10
**Kombucha**	
green tea	5
black tea	5
green & black teas	6
starter	1
commercial scoby	1

Table lists the number of samples with each set of contents, including substrates, brine, starter, or SCOBY.

Table S2. Nextera indexing primers

i7 Index Name	i7 Bases for Sample Sheet	i5 Index Name	i5 Bases for Sample Sheet
N701	TAAGGCGA	S501	TAGATCGC
N702	CGTACTAG	S502	CTCTCTAT
N703	AGGCAGAA	S503	TATCCTCT
N704	TCCTGAGC	S504	AGAGTAGA
N705	GGACTCCT	S505	GTAAGGAG
N706	TAGGCATG	S506	ACTGCATA
N707	CTCTCTAC	S507	AAGGAGTA
N708	CAGAGAGG	S508	CTAAGCCT
N709	GCTACGCT	S510	CGTCTAAT
N710	CGAGGCTG	S511	TCTCTCCG
N711	AAGAGGCA	S513	TCGACTAG
N712	GTAGAGGA	S515	TTCTAGCT
N713	GTAGAGGA	S516	CCTAGAGT
N714	GCTCATGA	S517	GCGTAAGA
N715	ATCTCAGG	S518	CTATTAAG
N716	ACTCGCTA	S520	AAGGCTAT
N718	GGAGCTAC	S521	GAGCCTTA
N719	GCGTAGTA	S522	TTATGCGA
N720	CGGAGCCT	D501	TATAGCCT
N721	TACGCTGC	D502	ATAGAGGC
N722	ATGCGCAG	D503	CCTATCCT
N723	TAGCGCTC	D504	GGCTCTGA
N724	ACTGAGCG	D505	AGGCGAAG
N726	CCTAAGAC	D506	TAATCTTA
N727	CGATCAGT	D507	CAGGACGT
N728	TGCAGCTA	D508	GTACTGAC
N729	TCGACGTC		
D701	ATTACTCG		
D702	TCCGGAGA		
D703	CGCTCATT		
D704	GAGATTCC		
D705	ATTCAGAA		
D706	GAATTCGT		
D707	CTGAAGCT		
D708	TAATGCGC		

Supplemental Figures

A

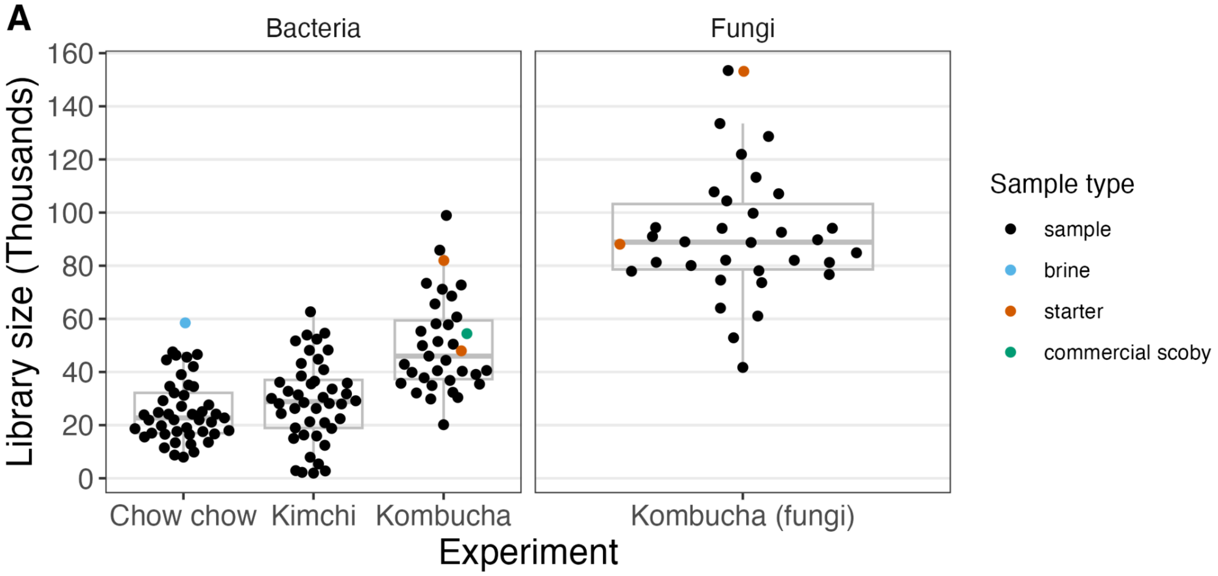

B

Experiment	Minimum library size
Chow chow	7,959
Kimchi	1,940
Kombucha	20,156
Kombucha (fungi)	41,724

Figure S1 Library sizes. A) Library sizes of samples in each experiment. Number of reads in samples after filtering. B) Minimum library size in each experiment. Samples were rarefied to this read depth for alpha diversity analyses.

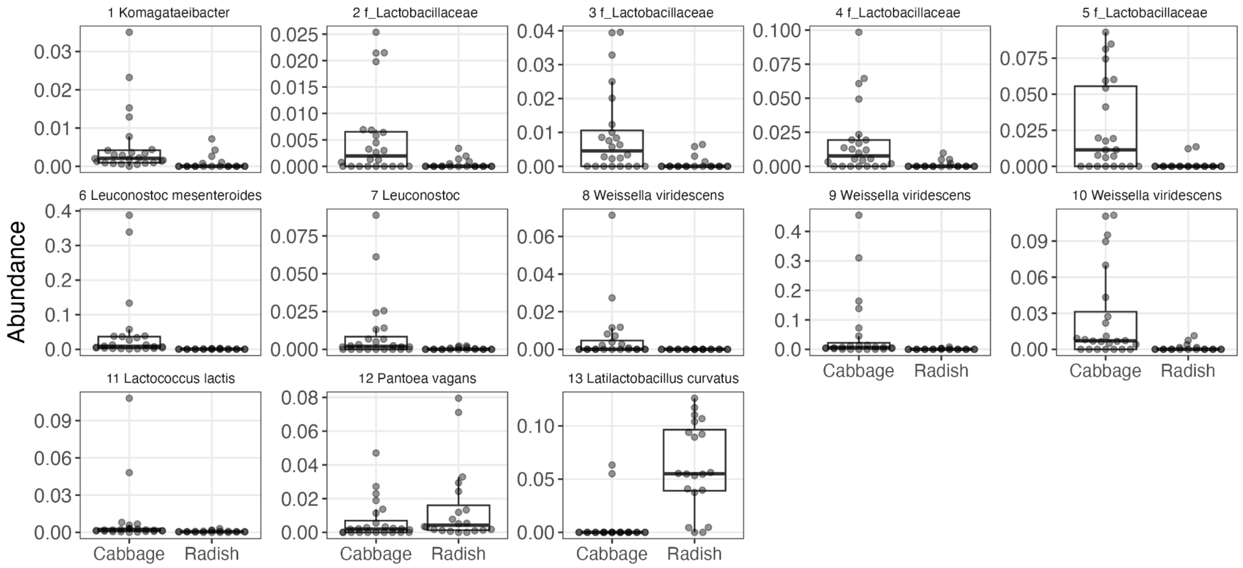

Figure S2. Relative abundances of significant indicator ASVs. Relative abundances in each sample of the 11 ASVs found to be indicators for cabbage as a kimchi substrate, and 2 ASVs found to be indicators of radish as a substrate using the indicspecies package (FDR corrected $p < 0.05$).

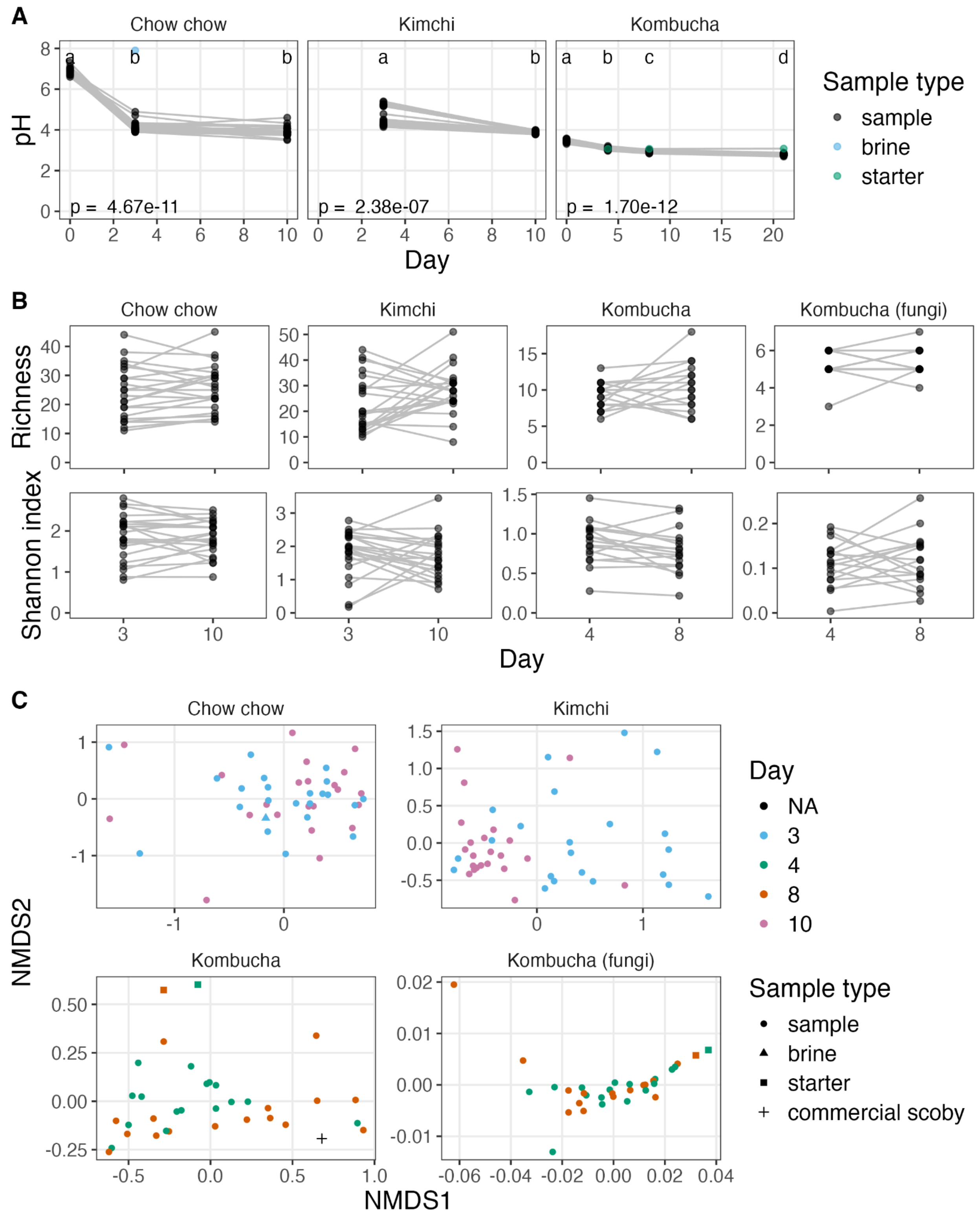

Figure S3. Community succession over time. A) Sample pH over time. *p*-values of Kruskal-Wallis (chow chow and kombucha) and Wilcoxon rank sum test (kimchi) are shown. Results from Dunn's post ßhoc test with Benjamini-Hochberg *p*-value adjustment are shown by letters. Kimchi pH was compared

using Wilcoxon rank sum. Brine and starter samples were plotted but not included in statistical analysis. B) Alpha diversity, measured as observed richness and Shannon index, by sampling day. Samples were rarefied to the minimum library size in each respective experiment (see Figure S1). Paired Wilcoxon ranked sum tests were used to compare observed richness and Shannon indices between the first and second days of sample collection. There were no significant differences between observed richness and the Shannon index between the first and second measurements, indicating that alpha diversity did not change over time as fermentation progressed. Note that axes differ among panels. C) Beta diversity in samples over time. Non-metric multidimensional scaling on Bray-Curtis distances of ASV relative abundances. PERMANOVA tests on Bray-Curtis distances demonstrated that chow chow (p = 0.007) and kimchi (p = 0.009) communities varied, but not kombucha bacteria (p = 0.963) or fungi (p = 0.689). Samples were not rarefied before analysis. Brine, starter, and commercial scoby samples are plotted in the NMDS but were not assessed in PERMANOVA analysis.

Supplemental Materials

<u>Supplementa Materials 1. Chow chow Recipe</u>

Fermenting Vegetables

- Decide how you want your vegetables prepared, and shred, chop, or leave your vegetables whole.
- Layer your vegetables in your fermentation vessel - food-grade plastic, ceramic, or glass (not metal).
- Add kosher or sea salt (non-iodized) as you go - salt to taste (about 1.5 tsp/lb).
- You can add spices or other flavorings.
- If you wish for your vegetable to ferment in its own juices (like sauerkraut), stomp or squeeze the vegetable until it releases enough juice for the vegetable to be **completely submerged**.
- If you wish to cover your vegetable with a brine solution, pour non-chlorinated water over salted vegetable and shake until salt dissolves. Make sure vegetables are **completely submerged** under the brine.
- Optional: weigh your vegetables down with a piece of mesh and/or a weight so that they stay under the liquid.
- Sealing Options:
    - Cover your vessel with a cloth, secured with a string or band.
    - Close your vessel loosely with a lid.
    - Seal your vessel tightly with a screw-top lid, and burp it daily.
    - Seal your vessel with an airlock.
- Leave to ferment in a cool dark place (ideally 50-70 degrees F).
- Enjoy at any stage (4-6 weeks for maximum beneficial bacteria)!

Supplemental Materials 2. Kimchi Recipe:

Mama Kwon's Kimchi

INGREDIENTS

- 1 head of large napa cabbage (around 3 to 4 lbs)
- 1 cup of Korean Coarse Sea Salt
- 1 gallon of water for salting
- 1 medium sized Korean radish (1 to 2 lbs) – peel and cut into 1" pieces
- 1 bunch of green onions cut into 1 inch pieces (6 to 8 stalks)
- 1 head of garlic (peeled)
- 1 piece of peeled ginger (about 1" size)
- 1 medium sized onion peeled and chopped into large pieces
- 1 Red bell pepper seeded and chopped into large pieces
- ¼ cup of Korean salted shrimp
- ¼ cup of fish sauce
- ¼ cup of Korean Anchovy Sauce
- 2/3 cup of Korean Red Pepper Powder for Kimchi (gochugaru)
- 2 tsp of sugar

INSTRUCTIONS

1. You will need 2 large mixing bowls and 1 medium sized bowl.
2. Cut napa cabbage into quarters
3. Mix 1 cup of Korea Coarse Sea Salt into 1 gallon of water in a large mixing bowl (water should be super saturated with salt and normal for all the salt to not melt).
4. Soak the quartered cabbage in the sea salt solution and set aside in another large mixing bowl (leaves should be facing up so salt water will not drain out – see image). Pour the remaining salt solution over the cabbages. Rinse the bowl to be used later.

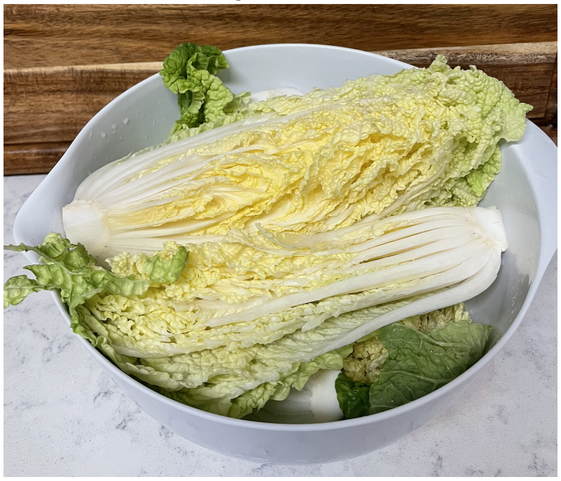

5. Let cabbage salt for about 3 to 4 hours until leaves are nicely salted and wilted (bend the white part of the leaf to see if it snaps or not to see if wilted). If leaves snap instead of bending, sprinkle some sea salt over the cabbages and let it sit for another hour.
6. In the meantime, blend the garlic, ginger, onion, and bell pepper in a food processor until nicely blended) and place in a medium sized mixing bowl.
7. Mix the chopped green onions with the blended garlic/ginger/onion/bell pepper mixture and set aside.
8. Rinse and clean the salted cabbage.

9. Chop the salted cabbage into 1” pieces and place in the large mixing bowl.
10. Add the cut Korean radishes to the cabbage and toss together.
11. Add the Korean Red Pepper Powder into the cabbage/radish mix and coat evenly.
12. Add the Korean salted shrimp, fish sauce, and Korean anchovy sauce to the cabbages/radish mix and make sure they all get mixed well together.
13. Finally add sugar and the garlic/ginger/onion/bell pepper/green onions to the mix and mix all of it together.
14. Taste to see if more fish sauce/anchovy sauce is needed and add more if desired. Kimchi should be moderately salty to taste.
15. Store kimchi into glass jars (plastic jars are okay with you only have plastic jars). Do not fill to the top. Leave around 1 inch space from top.
16. Pour 1/2 cup of drinking water into the mixing bowl that kimchi was made in and ‘rinse’ the bowl. Pour the water mixture into the jar until it covers kimchi. Close the lid and let it ferment at room temperature 2 to 3 days.
17. Once fermented to desired taste, store in the refrigerator.

Supplemental Materials 3. Kombucha Recipe

Kombucha

Kombucha is an ancient fermented tea, it is first recorded around 212 B.C. during the Chinese Tsin dynasty but most likely dates back further. It was hailed as the “tea of immortality”. Kombucha is a tart, slightly sweet and refreshing ferment. It is loved by many for its healing and energizing properties.

This recipe comes from research and trial and error. After you get the basic technique down, experiment and find what YOU like best! Kombucha is versatile and unique.

You will need:

- Glass jars to the desired size of brewing (for purposes of this recipe, measurements will be for a 1⁄2 gallon brew).
- Coffee filters or cloth napkins and rubber bands
- Purified water (no distilled or RO water due to lack of vitamins and minerals your SCOBY needs).
- Organic sugar or raw sugar (my preference any sugar will work)
- Organic green tea and organic black tea (organic is my preference)
- Brewing Culture (also called a “mother” or SCOBY)
- One cup of starter tea (mature kombucha)

Method:

- Heat 6-7 cups of water to just boiling (not a rolling boil, just until there are bubbles forming at the bottom/sides of the pot)
- Add your sugar and stir
- Remove from heat and put in your tea bags – 2 green and 2 black. Cover the pot and allow the tea to steep for 10 to 15 minutes.
- Allow to cool to 70 F (Room temperature) hot temperatures will kill your scoby.
- Pour 1⁄2 cup to one cup of starter and your brewing culture into your brewing vessel and then
- add your sweet tea. (In the warmer months 1⁄2 cup in cooler months 1 cup- the more starter you use, the faster and sharper the kombucha will brew)
- Cover with a cloth/filter and rubber band.
- Allow to ferment undisturbed for 7-10 days or longer if you desire a longer fermentation.
- You can start tasting around day 4/5 with a straw. Taste daily to your deserved flavor/ferment.
- **Reserve completed kombucha (1 cup) for your next brew.**
- You can drink raw or move to Method part II for secondary flavoring.

Method Part II:

- You can drink raw or add flavor and complete a secondary ferment by adding your choice of fruit /flavoring to your completed kombucha and fermenting in an air tight container for another 3 or so days. Leave a small amount of headspace.
- If you do not want carbonation in your secondary ferment, place in a container covered with a loose fitting lid.

*****WARNING***** THE BOTTLES ARE UNDER PRESSURE AND HAVE THE POTENTIAL

TO EXPLODE. ALWAYS PLACE BOTTLES IN A SAFE PLACE, SUCH AS A COOLER OR OTHER ENCLOSED CONTAINER.

Important Information:

- ****DO NOT FORGET*** reserve one cup/completed mature kombucha from this completed brew to use as starter for your next brew
- Rolling boil water = flat water which equals flat brew
- Kombucha culture does not like fluoride or chlorine
- **Please place your bottles during a secondary ferment into a cooler and burp the bottles if you desire. The contents are under pressure during a second ferment and the bottles can explode resulting in injury.**

Congratulations! You have successfully brewed great tasting Kombucha!

Chart:

- One quart- 3 cups of water- 1⁄2 TSP Loose leaf tea or 2 bags- 1⁄4 cup of sugar and 1⁄4-1/2 cup of starter
- Gallon- 13-14 cups of water-2 TBL Loose leaf or 8 tea bags- 1 cup of sugar – 1-2 cups of starter

*****These statements & recipe are not intended to cure or treat illness or disease, Southern Peak Brewery makes no claim to cure any illness or disease*****

*****Always ferment responsibly, Kombucha bottles can explode and also have the potential to contain up to 2% alcohol*****

Southern Peak Brewery
950 Windy Road, Suite 100 Apex, NC 27502
www.southernpeakbrewery.com

Supplemental Materials 4. Reflections

"The whole project, itself, sparked thoughts for me [Sarah] with regards to historical brewing of kombucha and questions about the purpose of that seal that forms as the brew matures. People think you have to add a piece of SCOBY into every new brew, but you don't – a new pellicle spontaneously forms every time you brew a new batch of kombucha, as long as you add mature kombucha starter. That spontaneous formation draws in environmental microbes – which means that your environment and your inputs matter and make a difference to your product. In times when water was often not safe to drink, that pellicle (SCOBY seal) may have been critical for sealing the kombucha and preventing pathogens from getting into the beverage – and, importantly, the form and function of that seal has not changed – even though our fermentation methods have changed over time. For example, we have shifted from using honey to using refined sugars to ferment tea – but the spontaneous formation and the function of the pellicle itself has not changed. But what about the microbes and matter that make up the pellicle? That ecological variance likely reflects aspects of your environment, including whether you open your windows or have pets or wear your shoes inside. As a chef, the readouts were interesting but I'm still missing how the results can be useful in real life. How we share cultures and how we engage with the public matters, and it happens more than people think – and that represents an untapped opportunity to share how your environment and what you put into your ferment matters. I would have loved to have included a starter from a different environment – from across town or from another state – so we could have a discussion about how they differ, and how your environment matters.

I love that the ferments were kept in the window, so we could go back to the Museum and check on them and watch how the pellicles grew in each jar over time – but I wish we had had the added piece of the data updates, so we could connect the numerical findings back to what we were seeing in the jars. My kombucha workshop was attended by teachers, so linking the data to the aspects that we experience in the ferments (smell, sight, taste) would have been a powerful addition to make science more relatable to the public. Something I loved about the workshop was that it involved so many educators, from all across the state, who brought so much enthusiasm to learn new ways to make science modern and relatable. In some ways we've also taken the science aspect out of our food – because we are a culture of convenience – and kombucha and fermentation helps us bring elements of science and art back to our food.

I would also have loved to meet back, for a workshop report-back to share the pH and sequencing results, so we could all learn and appreciate the results and why they are important for the product and the nutritional and health impacts of the food."

- Sarah Michalski with Erin A. McKenney

"Not having much prior experience teaching science to a class or workshop, I learned how to organize such an event, collaborate with my colleagues and the chefs on the food preparations and scientific presentations. I not only learned from the chefs how to make these dishes, but historical aspects and details of their familiar past that influence their fermentation practices.

From the participants' survey responses following the first workshop, kimchi, we implemented suggestions for future workshops, such as creating the kombucha fermented tea workshop and adding small groups, more prepping ahead of time and having available an assembly line of ingredients. I was encouraged by small "ah-ha" moments during the workshops, when both the chefs and participants realized that microbial organisms, specifically the lactic acid producing bacteria, were the workhorses and what biochemical pathways were involved in preserving these types of foods or tea."

- Christina E. Roche

"My discussions with bakers have helped highlight experts' curiosities and gaps in knowledge or understanding, and have informed hypothesis development and experimental design for sourdough studies (for example, to study the effects of flour on succession dynamics). In the fermented foods workshops, Soo Kwon, Elizabeth Weichel and Amanda Matson expressed curiosity about the microbes that make their fermentations come alive. In our experience, unless they have specific training in food science, even professional fermenters often do not fully grasp the complexity or nuance of the diversity of taxa and metabolic processes that microbes contribute to the foods they produce – much less how much or little is understood about those dynamic processes."

- Erin A. McKenney
